# Comprehensive treatment of diabetic endothelial dysfunction based on pathophysiological mechanism

**DOI:** 10.3389/fmed.2025.1509884

**Published:** 2025-02-28

**Authors:** Zhao Liu, Jun Lu, Wenjun Sha, Tao Lei

**Affiliations:** ^1^Department of Endocrinology, Putuo Hospital, Shanghai University of Traditional Chinese Medicine, Shanghai, China; ^2^Shanghai University of Traditional Chinese Medicine, Shanghai, China

**Keywords:** diabetes mellitus, cardiovascular disease, endothelial dysfunction, oxidative stress, insulin resistance, antidiabetic agents, traditional Chinese medicine

## Abstract

Vascular endothelium is integral to the regulation of vascular homeostasis and maintenance of normal arterial function in healthy individuals. Endothelial dysfunction is a significant contributor to the advancement of atherosclerosis, which can precipitate cardiovascular complications. A notable correlation exists between diabetes and endothelial dysfunction, wherein chronic hyperglycemia and acute fluctuations in glucose levels exacerbate oxidative stress. This results in diminished nitric oxide synthesis and heightened production of endothelin-1, ultimately leading to endothelial impairment. In clinical settings, it is imperative to implement appropriate therapeutic strategies aimed at enhancing endothelial function to prevent and manage diabetes-associated vascular complications. Various antidiabetic agents, including insulin, GLP-1 receptor agonists, sulfonylureas, DPP-4 inhibitors, SGLT2 inhibitors, *α*-glucosidase inhibitors, thiazolidinediones (TZDs), and metformin, are effective in mitigating blood glucose variability and improving insulin sensitivity by lowering postprandial glucose levels. Additionally, traditional Chinese medicinal compounds, such as turmeric extract, resveratrol, matrine alkaloids, tanshinone, puerarin, tanshinol, paeonol, astragaloside, berberine, and quercetin, exhibit hypoglycemic properties and enhance vascular function through diverse mechanisms. Consequently, larger randomized controlled trials involving both pharmacological and herbal interventions are essential to elucidate their impact on endothelial dysfunction in patients with diabetes. This article aims to explore a comprehensive approach to the treatment of diabetic endothelial dysfunction based on an understanding of its pathophysiology.

## Introduction

Diabetes mellitus (DM) is a prevalent endocrine disorder characterized by a complex syndrome of metabolic dysregulation, primarily manifested as hyperglycemia owing to inadequate insulin secretion or impaired insulin action (1). The global burden of diabetes is significant, with a pooled analysis indicating an increasing prevalence among adults worldwide. According to the Diabetes Federation, the number of adults diagnosed with diabetes increased from 108 million in 1980 to 463 million in 2019 (2). Individuals with diabetes are at elevated risk of both microvascular and macrovascular complications. Cardiovascular diseases (CVDs) are the predominant contributors to morbidity and mortality in this population (3). Microvascular complications including neuropathy, nephropathy, and retinopathy significantly impair the quality of life of patients with DM. Conversely, macrovascular complications such as coronary heart disease, cerebrovascular disease, and peripheral arterial disease are the leading causes of mortality among diabetic individuals. A systematic review indicated that CVDs affect approximately 32.2% of patients with type 2 diabetes mellitus (T2DM) are affected by CVDs. Among T2DM patients with a mean age of 63.6 ± 6.9 years, 9.9% succumbed to CVDs, which accounted for 50.3% of all diabetes-related deaths (4). Endothelial dysfunction has been implicated in the pathophysiological mechanisms underlying diabetes and CVDs (5). It is essential to elucidate the pathogenic mechanisms linking these factors in order to inform therapeutic strategies. Consequently, this review aimed to investigate the comprehensive management of diabetic endothelial dysfunction, grounded in the pathophysiological interplay between DM and endothelial function.

## Retrieval strategy and method

PubMed, Cochrane Library, MEDLINE, CNKI, Wanfang, and VIP databases were searched for studies related to endothelial dysfunction in DM. The search terms included diabetes mellitus, DM, endothelial dysfunction, hypoglycemic agents, antidiabetic agents, and traditional Chinese medicine. The first 172 articles were retrieved and included, of which 42 were randomized controlled trials (RCTs) and 8 were systematic reviews. Other studies include literature reviews, guidelines, and other non-RCT types of experimental and clinical studies.

### What is vascular endothelial function?

To understand endothelial dysfunction, it is essential to first delineate the concept of endothelial function. Human blood vessels are structured in three distinct layers: the vascular endothelium (intima), smooth muscle cells (media), and surrounding elastic and connective tissue (adventitia). The vascular endothelium, which constitutes the innermost layer of the blood vessels, functions as an endocrine organ that secretes a diverse array of vasoactive substances. Upon stimulation with acetylcholine (ACH), endothelial cells release endothelium-derived contracting factors (EDRF) (6), which are ultimately identified as nitric oxide (NO) (7). Subsequent research has demonstrated that NO plays a critical role in regulating various physiological and pathophysiological processes, including neurotransmitter transmission, male erectile function, oxidative stress, and inflammatory responses (8). In addition to NO, endothelial cells are capable of synthesizing and releasing prostacyclin (PGI2) and endothelium-derived hyperpolarizing factors (EDHFs), both of which are significant EDRFs that modulate vascular tension (9). Endothelium-derived contractile factors encompass superoxide anion, endothelin-1 (ET-1), prostaglandin F2α (PGF2α), and thromboxane A2 (TXA2) (9). Furthermore, the renin-angiotensin system (RAS) serves as a crucial mechanism for regulating vascular tension owing to the high expression of angiotensin receptors in both endothelial and smooth muscle cells (10). Under normal physiological conditions, vascular tension is maintained by a balance between vasodilatory and vasoconstrictive factors. Endothelial dysfunction is characterized by the inability of endothelial cells to sustain vascular homeostasis, resulting from an imbalance between proatherogenic and antiatherogenic factors derived from the endothelium. This imbalance favors atherogenic factors, thereby contributing to the onset and progression of atherosclerosis. NO released from the endothelium exerts numerous anti-atherosclerotic effects, including vasodilation, inhibition of vascular smooth muscle cell proliferation, and suppression of leukocyte and platelet adhesion and aggregation. Consequently, endothelial dysfunction is typically associated with an increase in NO inactivation or a decrease in NO production by the endothelium, leading to the diminished bioavailability of NO.

### Morbidity mechanism of endothelial dysfunction in diabetes mellitus

**Figure 1:**
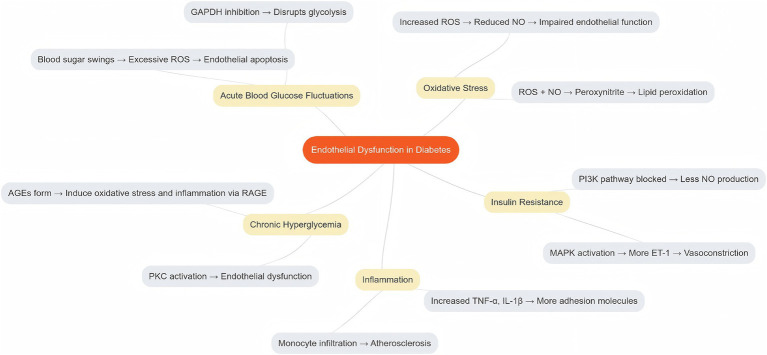
Morbidity mechanisms of endothelial dysfunction in DM.

DM is associated with endothelial dysfunction, as evidenced by various studies (11–13). Clinical investigations have revealed a reduction in endothelial function, evaluated through endothelium-dependent vasodilation, in individuals diagnosed with DM (14, 15). Prior research indicates that the primary contributors to endothelial dysfunction in DM are oxidative stress and inflammation, with significant disruptions occurring in the NO production and activation pathways (16). Although the precise mechanisms underlying the development of endothelial dysfunction in DM remain incompletely understood, existing studies have identified several potential mechanisms that elucidate the relationship between DM and endothelial dysfunction, as summarized in Table 1 and illustrated in Figure 1.

**Table 1:** Morbidity mechanisms of endothelial dysfunction in DM.

Morbidity mechanism	Specific description	References
Oxidative stress	The relationship between endothelial function and oxidative stress is significant, with insulin exerting its effects on the endothelium via the Ras-MAPK and PI3K-Akt-eNOS signaling pathways. In instances of insulin resistance, these pathways become impaired, leading to vascular endothelial dysfunction and the development of diabetic complications.	(16–25)
Selective insulin resistance	In the context of T2DM-associated insulin resistance, there exists a specific impairment in the PI-3 K signaling pathway. This defect contributes to the development of hyperinsulinemia, which in turn enhances the activation of MAPK signaling.	(26–32)
Inflammation	The correlation between insulin resistance and inflammation suggests that inflammation negatively impacts the PI-3 K/Akt signaling pathway responsible for NO production, leading to a decrease in NO bioavailability. This diminished availability of NO may undermine its substantial anti-inflammatory properties, consequently intensifying the development of inflammatory atherosclerotic lesions.	(33–43)
Chronic hyperglycemia	Chronic hyperglycemia, a defining characteristic of DM, is believed to play a significant role in the development of endothelial dysfunction through four primary mechanisms: the activation of PKC, the stimulation of the hexosamine and polyol pathways, and the formation of AGEs.	(44–47)
Acute glucose	Cellular metabolic adaptation to the toxic effects induced by elevated glucose levels may be enhanced by sustained high glucose concentrations, which create a continuous feedback mechanism. Conversely, intermittent exposure to high glucose may hinder the ability of cells to adapt to these toxic effects. In the absence of this continuous feedback, hyperglycemia can result in toxic consequences, characterized by increased oxidative stress and subsequent endothelial dysfunction.	(48–55)

### Oxidative stress

The vascular endothelium exhibits heightened sensitivity to fluctuations in systemic glucose levels, which distinguishes it from other tissues and cells that remain unaffected by such abnormalities. Consequently, the vascular endothelium may serve as a primary target for the detrimental effects of hyperglycemia (17). Reactive oxygen species (ROS) generated from molecular oxygen play a significant role in this context. Oxidative stress arises when there is an imbalance between the antioxidant defence system and ROS, favoring the latter. When the capacity of antioxidant systems to counteract ROS is inadequate, the harmful consequences of ROS become evident, including the disruption of signaling pathways and normal cellular functions through damage to cellular lipids, proteins, or DNA (18). Oxidative stress has emerged as a critical factor that contributes to vascular endothelial dysfunction, particularly in the context of chronic diseases and aging. There exists a reciprocal relationship between endothelial function and oxidative stress (19, 20).

ROS encompasses both radical species, such as the superoxide anion radical (O2^•−^), peroxy radicals, alkoxy radicals, and hydroxyl radicals, as well as non-radical species, including singlet molecular oxygen, hydrogen peroxide, organic hydroperoxides, hypochlorous acid, and ozone (20). The superoxide anion radical (O2^•−^) is generated by the removal of an electron from molecular oxygen and has a high affinity for NO, thereby diminishing its bioavailability. Furthermore, the direct interaction between NO and O2^•−^ (21) results in the formation of pernitrite, a potent oxidant that can induce lipid peroxidation, multi-step tyrosine nitration, DNA damage, and ultimately, cell death (21). Tetrahydrobiopterin (BH_4_), a crucial cofactor for endothelial NO synthase (eNOS), can be oxidized to its inactive form by peroxynitrite, leading to a reduction in BH_4_ availability. In instances of insufficient BH_4_, uncoupled eNOS generates O2^•−^ instead of NO (22), thereby establishing a close association between O2^•−^ and the progression of endothelial dysfunction. Once oxidative stress is established, endothelial function is perpetually compromised via a self-reinforcing cycle of increased O2^•−^ production.

Research indicates that elevated levels of free radicals can adversely affect the morphology and functionality of tissue membranes, contributing to the development of complications associated with DM (16). The primary mechanism underlying endothelial dysfunction (23–25) involves the uncoupling of ROS generated by xanthine oxidase (XO), cyclooxygenase (COX), and eNOS, which elevates ROS levels and leads to the inactivation of NO. Insulin plays a regulatory role in endothelial function by modulating protein kinase activity, NO production, and vasodilation through the Ras-MAPK and PI3K-Akt-eNOS signaling pathways. However, the onset of insulin resistance disrupts these pathways, resulting in vascular endothelial dysfunction and the manifestation of DM-related lesions.

### Selective insulin resistance

Beyond its function in glucose regulation, insulin resistance triggers intracellular signaling pathways that are essential for preserving endothelial health, with the phosphatidylinositol-3 kinase (PI-3 K)/Akt pathway being the most protective against vascular damage (26, 27). Conversely, activation of the mitogen-activated protein kinase (MAPK)/extracellular signal-regulated kinase (ERK) pathway enhances the expression of ET-1, resulting in detrimental effects on cellular proliferation (28). Under normal physiological conditions, the PI-3 K pathway is predominant in the regulation of vasomotor control (29). However, it is widely recognized that in the context of insulin resistance associated with T2DM, there exists selective impairment of the PI-3 K pathway, which remains unaffected by signaling through the MAPK pathway (30, 31). Furthermore, this selective resistance contributes to hyperinsulinemia, which in turn activates MAPK signaling (32).

## Inflammation

T2DM is characterized by a persistent systemic inflammatory state, with elevated levels of circulating inflammatory markers frequently observed in individuals with diabetes and obesity (33, 34). Vascular inflammation encompasses various cellular components, including inflammatory cells (neutrophils, lymphocytes, monocytes, and macrophages), endothelial cells, vascular smooth muscle cells (VSMCs), and extracellular matrix (ECM). In blood vessels, acute inflammation is marked by vasodilation, increased vascular permeability, and blood stasis. Alterations in the cytoskeleton of endothelial cells (ECs) lead to disruption of intercellular junctions, thereby enhancing vascular permeability. When ECs are subjected to a chronic inflammatory response induced by pro-inflammatory cytokines, including tumor necrosis factor (TNF), interleukin-1β (IL-1β), IL-6, and interferon-*γ* (IFN-γ), they can activate inflammatory cells, such as monocytes and T lymphocytes, prompting their chemotaxis, adhesion, and infiltration into the arterial intima, where they differentiate into macrophages. These macrophages subsequently engulf lipoproteins, resulting in the formation of foam cells that contribute to plaque development. The upregulation of adhesion molecules, including selectin, vascular cell adhesion molecule-1 (VCAM-1), and intercellular adhesion molecule-1 (ICAM-1), facilitates the adhesion of inflammatory cells to monocytes and promotes the recruitment of neutrophils, lymphocytes, and macrophages along with the release of additional cytokines. This cascade of events leads to ECM deposition, granulation tissue formation, connective tissue proliferation, increased cell adhesion, heightened permeability, and apoptosis, ultimately resulting in vasculopathy (35).

The levels of Advanced Glycation End Products (AGEs) are elevated in individuals with T2DM, contributing to the development of insulin resistance. The presence of AGEs, along with insulin resistance, can activate the nuclear factor kappa-light-chain-enhancer of activated B cells (NF-κB), a nuclear transcription factor. NF-κB, along with phosphoinositide 3-kinase (PI-3 K), p38 MAPK, and other signaling molecules, mediates intracellular signaling pathways that promote the release of various inflammatory cytokines, leading to endothelial cell dysfunction (36, 37).

Research utilizing obese animal models has demonstrated that adipose tissue serves as a source of inflammatory cytokines, which correlates with elevated plasma levels of TNF-*α* (38, 39). TNF-α is known to activate NF-κB (40). Additionally, NF-κB can be activated by free fatty acids and the receptor for advanced glycation end products (RAGE), both of which are prevalent in the diabetic milieu. Notably, obese mice with mutations that render the TNF-*α* gene inactive exhibit a marked increase in insulin sensitivity (41). This suggests that prolonged activation of NF-κB within cells positions it as a primary responder to various inflammatory stimuli.

The relationship between insulin resistance and inflammation indicates that inflammation negatively impacts the PI-3 K/Akt signaling pathway, which is responsible for the production of NO, ultimately leading to a decrease in NO bioavailability (42). Furthermore, the exposure of cultured ECs to TNF-*α* has been shown to impair the expression of endothelial nitric oxide synthase (eNOS) (43). Consequently, the diminished availability of NO may undermine its anti-inflammatory properties, thereby exacerbating the development of inflammatory atherosclerotic lesions.

### Chronic hyperglycemia

Chronic hyperglycemia, a defining characteristic of DM, is believed to play a significant role in the development of endothelial dysfunction through four primary mechanisms: activation of protein kinase C (PKC), stimulation of the hexosamine and polyol pathways, and formation of AGEs (44, 45).

Intracellular oxygen is predominantly generated by mitochondria (46). Pyruvate, which is synthesized in the cytoplasm through glycolysis, is subsequently utilized in mitochondria to produce adenosine triphosphate (ATP) via oxidative phosphorylation. Following its synthesis, pyruvate is transported into the mitochondria where it undergoes oxidation to yield water (H_2_O), carbon dioxide (CO_2_), nicotinamide adenine dinucleotide (NADH), and flavin adenine dinucleotide (FADH2) through the tricarboxylic acid (TCA) cycle (44). The electrons derived from mitochondrial NADH and FADH2 are utilized by the electron transport chain located on the inner mitochondrial membrane, which serves as the energy source for ATP synthesis. During this process, electrons are transferred through the mitochondrial electron transport chain, which concurrently facilitates proton pumping from the mitochondrial matrix into the intermembrane space. This proton pumping generates a proton gradient across the inner mitochondrial membrane, thereby promoting enhanced transport of NADH and FADH2 into the electron transport chain. Given that NADH and FADH2 function as electron donors, electron transfer and proton pumping through the electron transport chain are augmented, resulting in an increased proton gradient across the mitochondrial inner membrane. Consequently, the electron gradient and proton pumping mechanisms may be inhibited, leading to an increase in electron leakage from the electron transport chain, and subsequently, an increase in oxygen production within the mitochondria (44).

Glyceraldehyde 3-phosphate dehydrogenase (GAPDH) is a critical enzyme involved in the glycolytic pathway and plays a vital role in the regulation of glycolysis. Research indicates that GAPDH activity is partially suppressed due to the excessive generation of mitochondrial superoxide (O2^•−^) resulting from hyperglycemia. Consequently, the inhibition of GAPDH activity by mitochondrial superoxide contributes to the accumulation of glycolytic intermediates that precede GAPDH, thereby enhancing the flow of these upstream metabolites into the glucose overutilization pathway (44).

Elevated glucose flux through the polyol pathway leads to an increased consumption of NADPH, which is essential for the regeneration of reduced glutathione. Consequently, intracellular levels of reduced glutathione diminish as a result of increased glucose flux, further exacerbating NADPH consumption. Given that reduced glutathione serves as the primary intracellular antioxidant, this reduction contributes to an increase in oxidative stress within the cell, ultimately resulting in endothelial dysfunction.

Elevated glucose flux in the hexosamine biosynthetic pathway may contribute to endothelial dysfunction. In this pathway, fructose-6-phosphate is enzymatically converted to glucosamine-6-phosphate, which subsequently leads to an increase in UDP-N-acetylglucosamine. This metabolite is essential for various biochemical processes, including proteoglycan synthesis and O-linked glycoprotein formation. Increased levels of UDP-N-acetylglucosamine facilitate the modification of transcription factors, nuclear proteins, and cytoplasmic proteins through O-linked N-acetylglucosamine, resulting in significant changes in gene expression and protein functionality. An example is the O-acetylglucosaminylation of eNOS at the Akt phosphorylation site, which inhibits eNOS activity (47), thereby leading to a decrease in NO production and subsequent endothelial dysfunction.

Hyperglycemia triggers PKC activation through the elevation of diacylglycerol levels, which subsequently initiates a cascade of pathogenic effects, including a reduction in eNOS expression. This condition is associated with the increased expression of ET-1, increased levels of plasminogen activator inhibitor, augmented expression of transforming growth factor-beta, and activation of NF-κB and NADPH oxidase. Collectively, these alterations contribute to the development of endothelial dysfunction.

The elevated intracellular synthesis of precursors to AGEs leads to alterations in plasma and ECM proteins, in addition to functional modifications of intracellular proteins. The engagement of AGE receptors on ECs triggers the production of ROS and activation of nuclear factor kappa B (NF-κB), which contributes to endothelial dysfunction. Consequently, endothelial dysfunction is attributed to hyperglycemia-induced mitochondrial superoxide (O2^•−^) production as well as the inhibition of GAPDH activity by mitochondrial O_2_, resulting in a diversion of glycolytic flux from the conventional glycolytic pathway to an alternative metabolic route.

### Acute glucose

Plasma glucose levels in individuals without diabetes are typically regulated within a narrow range, whereas patients with DM experience significant and rapid increases in blood glucose levels during the postprandial period. Both experimental and clinical investigations have demonstrated that acute postprandial hyperglycemia in patients with DM can adversely affect endothelial function by elevating oxidative stress levels (48). Notably, intermittent hyperglycemia may pose a greater risk to ECs than sustained hyperglycemia does. *In vitro* research indicates that intermittent hyperglycemia leads to a higher rate of endothelial cell apoptosis through the activation of PKC and NADPH oxidase, in contrast to persistent hyperglycemia (49, 50). Furthermore, clinical studies have established a link between glucose fluctuations and impaired endothelial function through oxidative stress mechanisms. Monnier et al. reported a strong correlation between markers of glucose fluctuation and oxidative stress, whereas no significant relationship was found between oxidative stress markers and other glycemic indicators such as fasting plasma glucose and hemoglobin A1c (HbA1c) (51). Additionally, Torimoto et al. (52) found an inverse correlation between glucose fluctuations and endothelial function assessments. These results implied that fluctuations in glucose levels may detrimentally affect endothelial function by increasing oxidative stress. However, the precise molecular mechanisms underlying the relationship between glucose variability and increased oxidative stress remain poorly understood. It has been hypothesized that the metabolic adaptation of cells to the toxic effects of elevated glucose may be enhanced by sustained high glucose concentrations owing to persistent feedback mechanisms. Conversely, intermittent high glucose levels may not promote cellular adaptation to toxic effects associated with elevated glucose levels. In the absence of continuous feedback, hyperglycemia can induce toxic effects resulting in increased oxidative stress and subsequent endothelial dysfunction. While HbA1c is commonly utilized as a therapeutic marker for glycemic control, it primarily reflects average glucose exposure over time rather than fluctuations in glucose levels. Large randomized controlled trials employing HbA1c as a measure of glycemic control have not demonstrated the efficacy of intensive glycemic management in reducing cardiovascular events (53–55). Consequently, it is imperative to consider not only HbA1c and fasting plasma glucose levels but also postprandial glucose levels in efforts to safeguard the endothelium from oxidative damage associated with postprandial hyperglycemia.

### The role of epigenetics in endothelial dysfunction in diabetic patients

The mechanisms under discussion indicate that environmental factors, such as hyperglycemia induced by dietary choices, may lead to endothelial dysfunction. It is a common assumption that rectifying these imbalances would result in swift restoration of endothelial function; however, this assumption is misleading. Subsequent research conducted following the Diabetes Control and Complications Trial (DCCT) and the United Kingdom Prospective Diabetes Study (UKPDS) has introduced the concept of “metabolic memory.” This phenomenon suggests that the effects of prolonged or temporary fluctuations in blood glucose levels can persist long after normalization efforts have been implemented (56). Such findings not only illustrate that intensive glycemic control does not completely reverse the vascular complications associated with preexisting hyperglycemia, but also highlight that the advantages of rigorous therapeutic interventions can persist for many years, even when glycemic control deteriorates to typical suboptimal levels (57, 58). Collectively, these findings imply that DM, particularly hyperglycemia, may induce genetic modifications in the cellular phenotypes. Epigenetic research has elucidated the mechanisms by which gene expression can be influenced, thereby contributing to increased susceptibility to T2DM and CVDs (59). The epigenetic theory is underpinned by three interrelated pathways: histone modification, DNA methylation, and non-coding RNA mechanisms.

Post-translational modifications of histones within chromatin can influence the conformation of DNA, thereby facilitating or obstructing access to particular sites, which, in turn, can enhance or inhibit gene transcription. The regulation of histone modifications is mediated by various enzymes responsible for the addition or removal of these modifications (59). Numerous studies have focused on ECs, revealing that alterations in the expression and activity of these enzymes are linked to complications arising from DM. For instance, specific patterns of histone methylation have been correlated with a chronic inflammatory phenotype in ECs subjected to elevated glucose levels (60).

Altered patterns of DNA methylation have been associated with atherosclerosis (61) and ECs; however, there is a paucity of research on the role of DNA methylation in DM and its vascular complications (62). Covalent methylation of cytosine residues represents a stable modification of DNA that remains intact throughout the experimental procedures, thereby offering a robust foundation for future investigations (63). Similar to histone modifications, DNA methylation can result in persistent alterations in gene expression, which makes it imperative to elucidate the intricate mechanisms involved. Studies have indicated that high-fat diets in obese rats can lead to the hypermethylation of specific genes (64). Consequently, it is plausible to hypothesize that a similar phenomenon may occur in patients with DM, particularly with respect to endothelial function.

MicroRNAs (miRNAs) are short RNA molecules that regulate the expression of various target proteins by promoting mRNA degradation or inhibiting translation. In the context of ECs, *in vitro* investigations have identified a complex network of miRNAs that significantly influence processes such as proliferation and migration (notably miR-320 (65), miR-503 (66), and miR-221/222 (67, 68)), inflammation (including miR-10a (169) and miR-126 (170)), and angiogenesis (with a specific emphasis on miR-221/222 (69), miR-126 (70), miR-210 (71), and miR-21 (72)).

The miR-200 family, comprising five members, miR-200a, miR-200b, miR-200c, miR-141, and miR-429 (73), plays a significant role in oxidative stress-induced endothelial dysfunction and cardiovascular complications associated with DM and obesity (74–76). The miRNA family has garnered considerable interest in the fields of biology and medicine because of its involvement in various critical biological processes. Research indicates that the miR-200 family primarily regulates cellular behavior by inhibiting the translation of target genes or facilitating mRNA degradation. Specifically, the miR-200 family is implicated in the regulation of several fundamental cellular processes, including (1) cell proliferation, which governs the cell cycle and influences cellular growth (77); (2) cell differentiation, which directs the maturation of progenitor cells into specialized cell types (78); (3) apoptosis, which is essential for maintaining normal cellular turnover and for the removal of damaged or superfluous cells (79); and (4) epithelial-mesenchymal transition (EMT), a process that involves alterations in cell morphology and function that are critical for both embryonic development and cancer metastasis (79). The multifunctional characteristics of the miR-200 family make them a promising therapeutic target, particularly in the context of cancer and other diseases characterized by aberrant cell proliferation or transformation.

The miR-200 family, particularly miR-200c, has been implicated in oxidative stress associated with endothelial dysfunction in DM. MiR-200c exerts its effects by directly targeting SIRT1, eNOS, and FOXO1, leading to a reduction in NO levels and an increase in the acetylation of SIRT1 substrates, including FOXO1 and p53. Acetylation of FOXO1 subsequently inhibits its transcriptional activity on genes that are critical for cellular defense against oxidative stress, such as SIRT1 and ROS scavengers, including catalase and manganese superoxide dismutase. This inhibition results in elevated ROS production and promotes phosphorylation of the p66Shc protein at Ser-36, which further enhances ROS levels and suppresses FOXO1 transcription (80). This feedback loop contributes to the exacerbation of oxidative stress and is particularly pronounced under conditions of heightened oxidative stress, such as aging and ischemia, ultimately leading to endothelial dysfunction (81).

Endothelium-dependent NO-mediated vasodilation in individuals with diabetes is influenced by a reduction in both NO synthesis and stability. The presence of excessive ROS diminishes the half-life of NO by converting it to peroxynitrite, thereby resulting in decreased NO levels. Hyperglycemia has been shown to enhances ROS production via NADPH oxidase. Additionally, microRNA-200c (miR-200c) has been implicated in the increased generation of ROS (80), which indirectly compromises vasodilatory function in patients with diabetes, ultimately leading to endothelial dysfunction. Diabetes adversely affects both the quantity and quality of endothelial progenitor cells (EPCs), resulting in a delayed response of these cells to vascular injury. Furthermore, hyperglycemia can induce dysfunction in circulating angioblasts (CAC) and endothelial colony-forming cells (ECFC), thereby diminishing their migratory, secretory, and angiogenic capabilities. The accumulation of advanced glycation end products (AGEs) may also promote apoptosis and impair the migratory, adhesive, and secretory functions of CAC and ECFC cells. miR-200c may exacerbate damage to and repair of the vascular endothelium in diabetic patients by influencing the functionality of these related cell types (80). In summary, miR-200c plays a critical role in the onset and progression of endothelial dysfunction in DM by disrupting regulatory pathways, affecting vasodilation, and impairing cellular repair.

**Figure 2:**
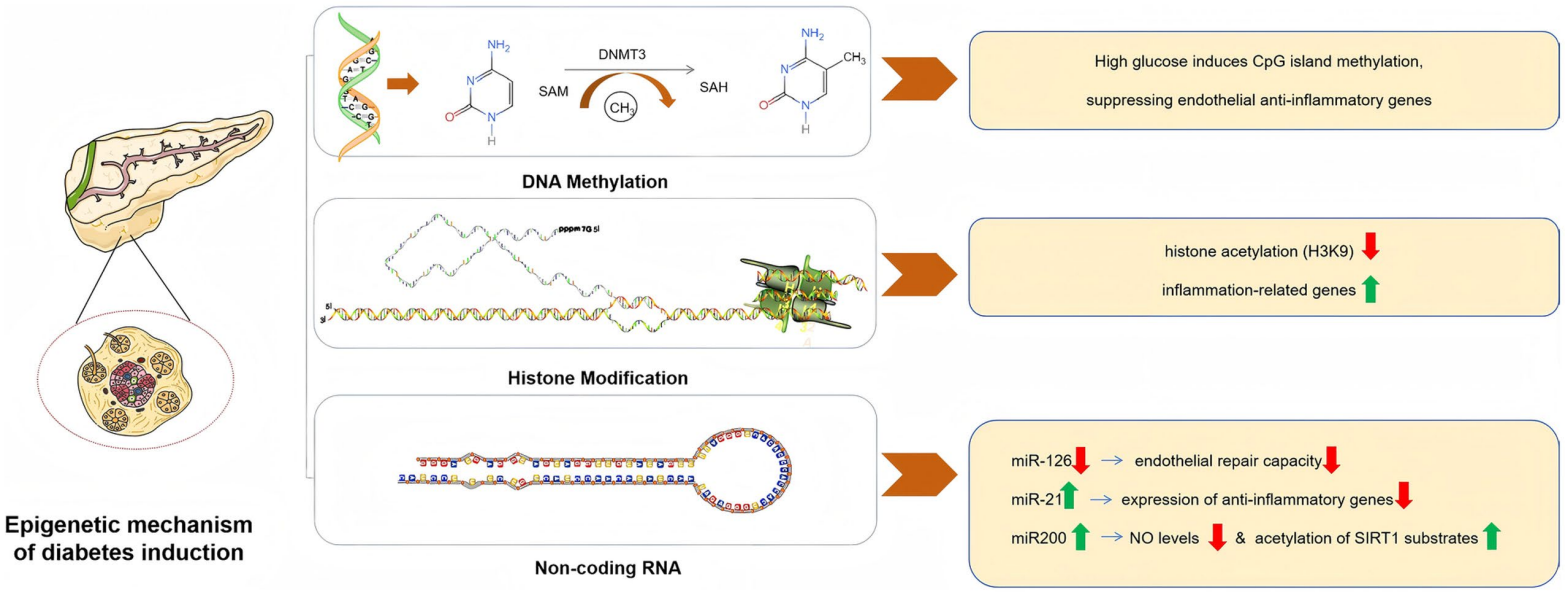
Epigenetic mechanism of diabetes induction.

Research has indicated that the effects of miRs can vary significantly under different physiological conditions. For instance, certain miRs are known to have protective and angiogenic effects (70, 71), whereas others exhibit detrimental anti-angiogenic properties (69, 72). Furthermore, it has been suggested that there is a distinct pattern of miRNA dysregulation in DM (82). Specifically, hyperglycemic environments may lead to excessive or insufficient expression of harmful or beneficial miRs, respectively, thereby contributing to vascular complications. An example is the observed upregulation of miR-320 in cardiomyocytes subjected to hyperglycemia, which correlates with diminished cell proliferation and migration (83). In healthy individuals, the levels of miRs in the plasma remain stable and consistent, positioning them as potential biomarkers for diseases such as T2DM (84). miRs may serve not only in monitoring disease progression but also in assessing the risk of future disease onset. Consequently, analyses of plasma from T2DM patients indicate that altered miR levels may play a role in the pathophysiological state of the disease (85). Despite recent progress in the understanding of epigenetic mechanisms related to DM and its complications, numerous questions remain. Additionally, the intricate nature of these interactions presents significant challenges for fully elucidating these mechanisms. (See Figure 2).

### Treatment of DM from the perspective of endothelial dysfunction

The selection of appropriate interventions to effectively enhance endothelial function is of considerable clinical importance in the management of cardiovascular events in patients with DM. However, the efficacy of commonly prescribed antidiabetic medications in improving endothelial function remains unclear. Flow-mediated dilation (FMD) is a non-invasive, endothelium-dependent method for evaluating endothelial function, typically employing ultrasound to measure alterations in the diameter of the brachial artery during ischemic conditions. An elevated FMD value is indicative of superior vascular elasticity (86). The expert consensus statement from the European Society of Cardiology advocates the utilization of FMD in the investigation of the pathophysiology of CVDs and, when feasible, in the identification of individuals at heightened risk for subsequent cardiovascular events (87). Numerous systematic reviews and meta-analyses have examined the impact of specific classes of antidiabetic medication on vascular function (88–90) (See Table 2).

**Table 2:** Effects of hypoglycemic drugs on endothelial function.

Drug category	Primary effect	Mechanism of action	Effectiveness	References
GLP-1R agonists	Anti-inflammatory, blood glucose reduction	Activates AMPK-eNOS pathway, promotes NO production, reduces inflammatory factors	High	(93–105)
Metformin	Enhances insulin sensitivity, reduces oxidative stress	Activates AMPK, inhibits NF-κB, reduces free radical production	High	(129–137)
Sulfonylureas	Increases insulin secretion	Stimulates insulin secretion, indirectly improves endothelial function	Moderate	(106–108)
*α*-Glucosidase inhibitors	Reduces glucose fluctuation, lowers oxidative stress	Inhibits carbohydrate breakdown, alleviates postprandial hyperglycemia-induced endothelial damage	Moderate	(123–125)
SGLT2 inhibitors	Improves glucose fluctuation and metabolic parameters	Protects endothelial function by reducing oxidative stress and inflammation	High	(112–122)
TZDs (Thiazolidinediones)	Reduces insulin resistance	Activates PPARγ pathway, decreases inflammation and oxidative stress	Moderate	(126–128)
DPP-4 inhibitors	Improves postprandial glucose fluctuation, partial anti-inflammatory effect	Inhibits DPP-4 enzyme activity, extends endogenous GLP-1 action duration	Low	(99–109)

Intensive glycemic control using insulin has been shown to mitigate both microvascular and macrovascular complications in individuals diagnosed with type 1 diabetes mellitus (T1DM) (91). In T1DM patients who maintain a healthy energy balance and exhibit no insulin resistance, insulin therapy may enhance vascular endothelial function, as selective insulin resistance is not a relevant factor. Conversely, the impact of insulin therapy on endothelial function in patients with T2DM appears to be contingent on the degree of metabolic control attained (92). Specifically, for obese or overweight T2DM patients experiencing insulin resistance due to overnutrition, the efficacy of insulin therapy on endothelial function is likely influenced by the level of metabolic control achieved by these individuals (92).

The Liraglutide Effect and Action in Diabetes: Evaluation of Cardiovascular Outcome Results (LEADER) (93) and the Trial to Evaluate Cardiovascular and Other Long-term Outcomes with Semaglutide in Subjects with Type 2 Diabetes (SUSTAIN-6) (94) have demonstrated a reduction in the risk of MACE, thereby supporting the cardiovascular benefits associated with glucagon-like peptide 1 receptor (GLP-1R) agonist therapy. Notably, GLP-1R agonists have been shown to significantly decrease arterial stiffness, as measured by pulse wave velocity (89). In a study conducted by Frías et al. (95), tirzepatide, which functions as a dual glucose-dependent insulinotropic polypeptide and GLP-1R agonist, exhibited a favorable impact on glucose homeostasis and was associated with a relatively rapid and pronounced antihypertensive effect. A subgroup analysis focusing on patients with hypertension in the trial may yield valuable insights. It is posited that the hypotensive effect of tizepatide is not contingent upon insulin regulation, and given that insulin has been shown to stimulate NO production and ET-1 secretion (96), its overall hemodynamic influence on blood pressure is minimal (97). Furthermore, insulin therapy has the potential to induce microcirculatory disturbances (98). A Bayesian network meta-analysis (99) identified GLP-1R agonists as the most effective antidiabetic agents to enhance FMD. Specifically, liraglutide significantly improved FMD in a subgroup of patients without CVDs compared with metformin and sulfonylureas. *In vitro* and animal studies have suggested that the effects of GLP-1R agonists on vascular endothelium can be categorized into direct and indirect mechanisms. Directly, GLP-1R agonists activate the AMPK-eNOS pathway in ECs, promoting NO production and facilitating endothelial vasodilation (100, 101). Indirectly, these agonists may decelerate atherosclerosis progression by providing endothelial protection through anti-inflammatory actions and enhancement of lipid metabolism (102–104). Additionally, GLP-1R agonists have been implicated in the inhibition of platelet aggregation and thrombosis, although it remains uncertain whether this mechanism is mediated by ECs (105). Notably, the impact of GLP-1R agonists on FMD appears to be more pronounced in a subgroup of patients with T2DM who do not have CVD (99). Consequently, some studies have hypothesized that individuals without CVD may exhibit heightened sensitivity to the effects of GLP-1R agonists, potentially due to more favorable changes in vascular function than those with CVD, or at least due to less severe impairment of endothelial function in this population.

Sulfonylureas function as insulin secretagogues; however, some studies indicate that the association between sulfonylureas and vascular endothelial function is not particularly strong (89). As a conventional antidiabetic medication, the cardiovascular implications of sulfonylureas in individuals with T2DM remain debatable (106). A meta-analysis encompassing a network of 18 studies involving sulfonylureas revealed that neither gliclazide nor glibenclamide was associated with an elevated risk of cardiovascular mortality, although glibenclamide was associated with an increased risk of cardiovascular death (107). This phenomenon may be attributed to their inhibitory effects on ATP-sensitive potassium channels in the cardiovascular smooth muscle cells. Such an effect can hinder myocardial ischemic preconditioning, leading to reduced coronary blood flow and increased peripheral vascular resistance (108).

Dipeptidyl peptidase 4 (DPP-4) and sodium-glucose cotransporter 2 (SGLT2) inhibitors represent two emerging classes of antidiabetic medications. The Saxagliptin Assessment of Vascular Outcomes Recorded in Patients with Diabetes Mellitus-Thrombolysis in Myocardial Infarction (SAVOR-TIMI) trial (99) was among the first completed studies evaluating DPP-4 inhibitors. This trial indicated that saxagliptin, a DPP-4 inhibitor, was associated with an increased hospitalization rate in patients with heart failure, thereby raising concerns regarding the cardiovascular safety of this pharmacological class (99). Subsequent randomized controlled trials (RCTs) focusing on MACE as the primary endpoint, such as the Sitagliptin Cardiovascular Outcomes Assessment Trial (TECOS), demonstrated no significant difference in the incidence of MACE between DPP-4 inhibitor treatment and placebo (109). Reaven et al. (110) noted that both SAVOR-TIMI and TECOS were primarily designed as non-inferiority trials, suggesting that their methodological frameworks lack sufficient power to adequately evaluate the cardiovascular benefits of DPP-4 inhibitors. Furthermore, two studies (104, 111) examining FMD from baseline to medium- and long-term follow-ups revealed that sitagliptin, a member of the DPP-4 inhibitor class, did not exhibit a discernible impact on endothelial function during the final observation period. The findings from the TECOS trial also indicated that DPP-4 inhibitors did not significantly decrease the incidence of MACE (109), implying that the protective effects of DPP-4 inhibitors on endothelial function remain limited.

SGLT2 inhibitors are pharmacological agents that lower blood glucose levels by obstructing renal reabsorption of glucose, thereby promoting its excretion in urine (112). The hypoglycemic action of these inhibitors is independent of the insulin levels. Studies have demonstrated that SGLT2 inhibitors effectively diminish postprandial blood glucose levels and overall glycemic fluctuations in individuals with DM (113, 114). Furthermore, these agents reduce insulin resistance and enhance peripheral insulin sensitivity (115–117). In addition to their glycemic effects, SGLT2 inhibitors also exert additional metabolic benefits, including reduction in plasma lipid concentrations, blood pressure, and body weight. Consequently, SGLT2 inhibitors may improve endothelial function by mitigating oxidative stress by reducing blood glucose levels in an insulin-independent manner, minimizing acute glycemic variations, enhancing insulin sensitivity, and improving various metabolic parameters. Several meta-analyses derived from small RCTs have indicated that SGLT2 inhibitors positively influence endothelial function, as assessed by FMD (89). However, it is important to note that SGLT2 inhibitors may elevate the risk of amputation in patients with T2DM and are significantly linked to peripheral vascular hypoperfusion (118). Additionally, clinical investigations have confirmed that SGLT2 inhibitors enhance endothelial function in individuals with DM (119–122).

A meta-analysis encompassing five RCTs investigating *α*-glucosidase inhibitor therapy indicated that these inhibitors effectively delayed the progression of carotid intima-media thickness (CIMT) in individuals diagnosed with T2DM (123). This finding implies that *α*-glucosidase inhibitors may exert beneficial effects on the vascular endothelial function. Postprandial elevation in glycemic levels is known to induce oxidative stress, which adversely affects endothelial cell functionality (124). Consequently, *α*-glucosidase inhibitors significantly mitigate postprandial hyperglycemia and reduce oxidative stress-related damage, thereby safeguarding the vascular endothelium. The Cardiovascular Evaluation Trial (ACE) involving acarbose, a member of the α-glucosidase inhibitor class, represents a completed RCT (125) that focused on this therapeutic category. However, the trial did not demonstrate a reduction in the incidence of heart failure or cardiovascular mortality in patients with T2DM or impaired glucose tolerance. It is important to note that this study had certain limitations, as it was conducted exclusively in China and the participant cohort consisted of individuals with impaired glucose tolerance and pre-existing coronary heart disease.

The impact of thiazolidinediones (TZDs), traditional insulinotropic agents, on cardiovascular events remains a subject of considerable debate, with previous RCTs yielding inconsistent findings. In a study utilizing a hypertensive rat model (126), pioglitazone (noting that rosiglitazone and troglitazone were withdrawn from first-line treatment and are therefore not addressed) was demonstrated to activate peroxisome proliferator-activated receptors (PPARs) through the modulation of ET-1 expression, which subsequently mitigated the effects of oxidative stress on vascular structures. Furthermore, pioglitazone has been shown to enhance vascular relaxation (126) by increasing the expression of ET-1 receptor B (ETB), facilitating the release of factors that promote endothelial cell relaxation. The PROspective pioglitazone Clinical Trial In macroVascular Events (PROactive trial) represents the first large-scale RCT evaluating the effects of pioglitazone monotherapy on cardiovascular outcomes. Findings from this trial indicated that pioglitazone significantly reduced all-cause mortality as well as the risk of nonfatal myocardial infarction and stroke in patients with T2DM and macrovascular disease (127). Additionally, a meta-analysis suggested that pioglitazone may have a beneficial effect on the risk of recurrent cardiovascular events in individuals with established CVDs (128). Nonetheless, it remains uncertain whether improvements in endothelial function attributed to pioglitazone translate into favorable outcomes regarding future cardiovascular events.

Metformin, recognized as an insulin sensitizer, has demonstrated efficacy in enhancing endothelial function, with a notable correlation between endothelial function and insulin resistance following treatment in individuals diagnosed with T2DM (129). Furthermore, metformin has been shown to enhances endothelial function in patients who do not have diabetes but exhibit insulin resistance (130). In a long-term RCT, the incorporation of metformin into treatment regimens significantly decreased the levels of various biomarkers indicative of endothelial function, which are associated with the morbidity risk of CVDs in T2DM patients, in comparison to a placebo group (131). *In vitro* studies also indicated that the beneficial effects of metformin may arise from multiple mechanisms, including the activation of adenosine 5′-monophosphate (AMP)-activated protein kinase (AMPK), endothelium-dependent vascular responses, and protection of ECs against oxidative stress (132). Preclinical investigations have revealed that metformin enhances endothelial function through phosphorylation of eNOS via AMPK activation (133, 134), activation of sirtuin-1 (135, 136), and promotion of antioxidant activity (132). Collectively, these findings suggest that metformin may improve endothelial function through a range of mechanisms, some of which may not be directly related to insulin resistance (133, 137).

### Effect of traditional Chinese medicine on diabetic endothelial dysfunction

As a significant component of complementary and alternative medicine, Chinese medicine plays a crucial role in addressing endothelial dysfunction associated with DM. Traditional Chinese medicine (TCM) is primarily employed for the management of endothelial dysfunction in DM, and various research findings have emerged in this area. Recent years have witnessed substantial advancements in the investigation of the individual components of TCM in the treatment of endothelial dysfunction related to DM (See Table 3).

**Table 3:** Effect of TCM on endothelial dysfunction in DM.

TCM name	Active component	Mechanism of action	Main findings	References
Tanshinone IIA	Lipophilic diterpenoids	Inhibits NADPH oxidase, reduces oxidative stress, enhances eNOS expression, and promotes NO production	Improves endothelial-dependent relaxation and endothelial function	(144, 145)
Berberine	Isoquinoline alkaloid	Inhibits NF-κB activity, reduces inflammatory responses	Protects against vascular damage and anti-atherosclerosis	(150–156)
Resveratrol	Polyphenol compound	Activates SIRT1, upregulates Nrf2, and reduces ROS production	Prevents endothelial cell aging, reduces oxidative stress	(139, 141, 142)
Puerarin	Isoflavone glycoside	Regulates NF-κB, inhibits NOX2 and NOX4-mediated oxidative stress	Protects the aorta of diabetic rats by reducing cell adhesion molecule expression	(146)
Curcumin	Polyphenolic compound	Blocks AMPL/p38 MAPK pathway	Protects endothelial cells from oxidative stress in diabetic rats	(138–140)
Matrine alkaloids	Sophora alkaloids	Activates MKK3/p38 MAPK/Nrf2 signaling pathway, reduces AGEs-induced apoptosis	Prevents apoptosis of diabetic endothelial cells	(143)
Astragaloside IV	Triterpene saponin	Modulates glucose/lipid metabolism, inhibits TGF-β1/Smad signaling, reduces oxidative stress	Protects aortic endothelial cells and inhibits NF-κB activation	(148, 149)
Danshensu + Paeonol	Phenolic acids	Inhibits p38 MAPK signaling, enhances BKCa protein expression in vascular smooth muscle cells	Reduces vascular endothelial cell apoptosis in diabetic conditions	(144, 145)
Quercetin	Flavonoids compounds	Increases the expression of ABCA1, ABCG1 and CYP7A1, promoted cholesterol efflux from macrophages, down-regulated the expression of p53, p21, p16 and ERK, enhanced autophagy to anti-apoptosis, and inhibited MCP-1 and inflammatory cytokines	Reduces ROS and releases NOS to protect endothelial cells	(157–162)

Research has demonstrated that turmeric extract can modulate plasma levels of endothelin, thromboxane, and prostaglandins, while mitigating vasospasm and enhancing both vasodilation and contraction in rats with T2DM over a treatment period of 16 weeks. The extract exerts its effects by inhibiting the activities of COX-2, NF-κB, and PKC as well as by altering the ratio of PGI2 to TXA2 in streptozotocin (STZ)-induced diabetic rats, thereby alleviating diabetes-induced vascular dysfunction. Additionally, turmeric extract inhibits the formation of glycated human serum albumin (CSA) through promoter activation. This treatment also leads to the induction and upregulation of IL-8 in VSMCs, which may obstruct the AMPL/p38MAPK signaling pathway and thereby protect endothelial cell function in T2DM rats (138–140).

Resveratrol inhibits the expression and upregulation of IL-8 in VSMCs induced by CSA (139). Furthermore, it activated nicotinamide adenine dinucleotide (NAD)-dependent sirtuin 1 in rats with T2DM exhibiting macroangiopathy over a 24-week period. This activation leads to deacetylation of sirtuin 1 target molecules, including NF-κB. Additionally, resveratrol enhances the expression of antioxidant enzymes through the activation of nuclear factor E2-related factor 2 (Nrf2) and reduces the activity of NADPH oxidase via established mechanisms, thereby inhibiting ROS production (141, 142).

Matrine alkaloids, which are bioactive compounds derived from *Sophora flavescens*, have been used to treat DM. Research indicates that these alkaloids can promote the phosphorylation of MAPK kinases, specifically MKK3 and MKK6, facilitating the nuclear translocation of Nrf2, enhancing the binding activity of antioxidant response elements, and increasing the expression of heme oxygenase and NADPH quinone oxidoreductase. Furthermore, matrine alkaloids have been shown to inhibit ROS production in aortic ECs and reduce endothelial cell apoptosis, both *in vivo* and *in vitro* (143). Consequently, it can be hypothesized that the MKKs/p38 MAPK/Nrf2 signaling pathway plays a significant role in the mechanism by which matrine alkaloids mitigate AGE-induced oxidative stress and subsequent apoptosis in diabetic ECs.

Tanshinone IIA reverses the uncoupling of eNOS induced by high glucose levels. This restoration occurs through the inhibition of several factors including NADPH oxidase, heat shock protein 90 (HSP90), GTP cyclin-1 (GTPCH1), dihydrofolate reductase (DHFR), and PI3K. Additionally, Tanshinone IIA ameliorated abnormal oxidative stress and enhanced endothelium-dependent relaxation in ECs of diabetic rats. The expression of eNOS is initiated at the transcriptional level, leading to increased production of NO, which serves a protective function against endothelial health (144, 145).

Puerarin was administered via intraperitoneal injection to rats with T2DM over a three-week period. These findings indicate that puerarin can modulate the NF-κB pathway and mitigate oxidative stress associated with NADPH oxidase isoforms NOX2 and NOX4. Consequently, this intervention resulted in the downregulation of cell adhesion molecule expression (146), thereby providing protective effects in the aorta of T2DM rats.

Hu Jing’s research revealed for the first time that the combination of danshensu and paeonol can markedly reduce the apoptosis of vascular ECs triggered by elevated glucose levels. This effect is mediated through inhibition of the p38 MAPK signaling pathway. Additionally, preliminary findings suggest that this combination may safeguard vascular smooth muscle function in diabetic arteries by upregulating BKCa protein expression (147).

These findings indicate that the protective effects of astragaloside on aortic ECs in rats with T2DM may be associated with the modulation of glucose and lipid metabolism abnormalities, alleviation of oxidative stress damage, and suppression of TGF-β1/Smad signaling pathways. Additionally, astragaloside appears to influence the expression of apoptosis-related genes, specifically Bcl-2, Bax, and Caspase-3 (148). Furthermore, it has been documented that both astragaloside IV and ferulic acid, administered at a dose of 50 mg/kg over a period of 10 weeks in diabetic rats, can inhibit the activation of the NF-κB signaling pathway by lowering blood glucose levels and decreasing oxLDL and TNF-*α* concentrations, demonstrating a synergistic effect (149).

Berberine is an isoquinoline alkaloid. Both traditional and contemporary medical research indicates that berberine exhibits a range of pharmacological properties, including hypolipidemic, antidiabetic, antitumor, anti-inflammatory, antidiarrheal, and antibacterial effects (150). Fatahian et al. (151) demonstrated that berberine may possess atheroprotective properties by lowering elevated plasma cholesterol levels, particularly low-density lipoprotein cholesterol (LDL-C), through mechanisms that are both dependent and independent of LDL receptors (LDLR). Additionally, berberine has been shown to inhibit macrophage migration and inflammatory responses, enhance endothelial cell function via its antioxidant properties (152–154), and suppress VSMC proliferation (155, 156).

Quercetin is an important flavonoid compound recognized for its pronounced anti-atherosclerotic properties. Contemporary research has demonstrated that quercetin exhibits potent antioxidant, anti-inflammatory, and antibacterial activities (157). Qian et al. (158) demonstrated that quercetin can diminish the release of ROS and nitric oxide synthase (NOS), thereby safeguarding ECs. Additionally, it enhances the expression of ATP-binding cassette transporters ABCA1 and ABCG1 as well as cytochrome P450 7A1 (CYP7A1), facilitating cholesterol efflux from macrophages. Quercetin also downregulates the expression of p53, p21, p16, and extracellular signal-regulated kinase (ERK) while promoting autophagy to counteract apoptosis. Furthermore, it inhibits monocyte chemoattractant protein-1 (MCP-1) and various inflammatory cytokines including interleukins IL-1, IL-2, IL-1β, IL-6, and TNF-*α* (159–162).

### Shortcomings and prospects

This study conducted a comprehensive evaluation of representative anti-diabetic medications and TCM for DM, revealing significant disparities in their effects on vascular function. Notably, GLP-1 receptor agonists, a class of novel anti-diabetic agents, appear to possess distinct advantages in enhancing vascular function in patients with DM. However, these studies have several limitations must be acknowledged in these investigations. For instance, the data extraction and transformation processes utilized in two of the included studies (101, 104) may introduce follow-up bias. Furthermore, while our analysis encompassed eight anti-diabetic drugs, variations may exist even among products within the same category. Additional factors contributing to the inconsistencies observed in this study include the duration of DM, which ranged from newly diagnosed cases to those exceeding three years, as well as racial and regional differences among the participants involved in the analysis. Moreover, although all included studies reported that the assessment of FMD was conducted by a professional sonographer in a blinded manner, the potential for measurement errors remains a concern.

TCM has been shown to have beneficial effects on blood glucose levels, insulin resistance, lipid metabolism, and oxidative stress through pathways involving AGE, PI3K/Akt, NF-κB, and AMP-AMPK, thereby ameliorating the endothelial dysfunction associated with DM. However, the existing body of research consists primarily of *in vitro* and animal model studies focusing on TCM extracts and their individual components, with a notable scarcity of human clinical trials. In comparison with contemporary antidiabetic pharmacotherapies, there is a limited amount of clinical trial data (163) evaluating the efficacy of TCM in addressing biomarkers of endothelial dysfunction in DM. Furthermore, TCM’s approach of TCM to disease treatment is predicated on syndrome differentiation, which involves tailoring prescriptions and therapies to specific syndromic presentations. Current in vitro and animal studies using TCM monomers and extracts do not adequately capture the principles of syndrome differentiation inherent in TCM practice, highlighting a significant gap in clinical research. Additionally, it is important to acknowledge that the indirect agonistic effects of certain TCM extracts, such as resveratrol and quercetin, are relatively weak, their bioavailability is insufficient, and there is a paucity of *in vivo* pharmacokinetic studies, which represents a limitation in the current research landscape.

Currently, our research group is engaged in investigating the treatment of endothelial dysfunction associated with DM using TCM. For instance, the study conducted by Dr. Xu et al. (164) utilized network pharmacology, molecular docking, and *in vitro* experimental validation to demonstrate that Danggui Liuhuang Decoction (DGLHD), a traditional Chinese medicinal formulation, effectively mitigates the release of pro-inflammatory factors and vascular endothelial growth factor by inhibiting the JAK2/STAT3 signaling pathway, thereby alleviating endothelial dysfunction in DM. Additionally, Dr. Sha et al. (who is also a co-author of this work) (165) discovered both *in vivo* and in vitro that Astragalus polysaccharide (APS) enhances vascular endothelial function in DM by activating the Nrf2/HO-1 pathway, which promotes macrophage polarization towards the M2 phenotype. Another investigation led by Dr. Sha (166) revealed that miR-142-3p contributes to the progression of diabetes by suppressing SPRED2-mediated autophagy, resulting in increased apoptosis, oxidative stress, and inflammatory cytokine secretion, which were ameliorated by resveratrol. Furthermore, Zhang et al. (167) identified a significant association between high-density lipoprotein cholesterol ratio (MHR) and endothelial dysfunction in T2DM in a cross-sectional study involving 243 patients, suggesting that MHR may serve as a novel biomarker for assessing vascular endothelial function. Wei et al. (168) reported that puerarin (Pue) mitigates endothelial cell injury and cardiovascular complications related to DM induced by LPS-ATP or high glucose through the ROS-NLRP3 signaling pathway. Along with my colleagues, Dr. Jin and Dr. Yu, I am also actively contributing to this field of research, and we are optimistic about achieving further advancements in the near future.

## Conclusion

Endothelial dysfunction is a significant therapeutic target in individuals diagnosed with DM. The pathophysiology underlying this dysfunction may be attributed to oxidative stress induced by acute glucose fluctuations, chronic hyperglycemia, and diminished NO production, resulting from selective insulin resistance in ECs. A multifaceted approach is recommended to address this issue, encompassing lifestyle modifications, such as weight reduction, engagement in aerobic exercise, and cessation of smoking. Furthermore, the use of antidiabetic medications that mitigate acute glucose variations, such as glinides, alpha-glucosidase inhibitors, and DPP-4 inhibitors, as well as those that enhance insulin sensitivity, including thiazolidinediones and metformin, are anticipated to positively influence endothelial function in patients with DM. Preclinical investigations have demonstrated that GLP1-R agonists, metformin, and SGLT2 inhibitors can enhance endothelial function through various mechanisms, some of which are independent of glycemic control or insulin signaling pathways, such as the activation of eNOS phosphorylation via AMP-AMPK and sirtuin-1. Careful selection of appropriate antidiabetic pharmacotherapy aimed at improving endothelial function is clinically important for the prevention of vascular complications associated with DM, thereby enhancing the overall prognosis for patients with this condition.

The pathophysiological mechanisms underlying morbidity associated with DM are intricate, and vascular endothelial dysfunction has received considerable attention in recent years. ECs are crucial components of the endocrine system and are particularly susceptible to environmental influences. Factors such as hyperglycemia, AGEs, oxidized low-density lipoprotein (ox-LDL), and abnormal insulin expression can directly or indirectly induce endothelial dysfunction. TCM holds significant potential in the clinical management of diabetic endothelial dysfunction owing to its multi-component and multi-target nature. Increasing recognition among pharmacological researchers has emerged regarding the efficacy of TCM monomers, extracts, and compounds for addressing endothelial dysfunction associated with DM. Recent studies have shown that TCM primarily modulates several signaling pathways, including AGEs, PI3K/AKT, NF-κB, Nrf2, LOX-1, and AMPK, to enhance NO bioavailability, inhibit angiotensin II synthesis, mitigate oxidative stress, regulate inflammatory and angiogenic factors, and prevent thrombosis. These actions contribute to the effective restoration of vascular ECs and attenuation of the onset and progression of endothelial dysfunction in DM.

However, the complex composition and numerous targets of TCM pose challenges in elucidating its mechanisms of action and the associated signaling pathways that ameliorate endothelial dysfunction in DM. Future research endeavors may benefit from integrating network pharmacology and proteomics to identify key genetic information and signaling pathways.

While this article aims to underscore a holistic approach to treating endothelial dysfunction in DM, it is noteworthy that the integration of Western medicine with TCM in the management of related diseases is actively practiced in China, yielding positive outcomes. Nonetheless, there remains a paucity of comprehensive clinical studies that are multi-centered, involve large sample sizes, and maintain high quality for the treatment of endothelial dysfunction in DM. This gap represents a focal point for our research group, which aspirates for more extensive, thorough, and in-depth experimental and clinical investigations within the medical community.

## Glossary

DM: Diabetes mellitus
CVDs: Cardiovascular diseases
T2DM: Type 2 diabetes mellitus
ACH: Acetylcholine
EDRF: Endothelium-derived relaxing factors
NO: Nitric oxide
PGI2: Prostacyclin
EDHFs: Endothelium-derived hyperpolarizing factors
ET-1: Endothelin 1
PGF2α: Prostaglandin F2α
TXA2: Thromboxane A2
RAS: Renin-angiotensin system
ROS: Reactive oxygen species
O2• −: Superoxide anion radical
BH4: Tetrahydrobiopterin
eNOS: Endothelial nitric oxide synthase
XO: Xanthine oxidase
COX: Cyclooxygenase
MAPK: Mitogen activated protein kinase
PI3K: Phosphatidylinositol-3 kinase
VSMCs: Vascular smooth muscle cells
ECM: Extracellular matrix
ECs: Endothelial cells
TNF: Tumor necrosis factor
IL-1β: Interleukin-1β
IFN-*γ*: Interferon-γ
VCAM-1: Vascular cell adhesion molecule-1
ICAM-1: Intercellular adhesion molecule-1
AGEs: Advanced Glycation End Products
NF-κB: Nuclear factor-κB
RAGE: Receptor for advanced glycation end products
ATP: Adenosine triphosphate
H2O: Hydroxylic acid
CO2: Carbon dioxide
NADH: Nicotinamide adenine dinucleotide
FADH2: Flavine adenine dinucleotide-reduced
TCA: Tricarboxylic acid
GAPDH: Glyceraldehyde 3-phosphate dehydrogenase
PKC: Protein kinase C
HbA1c: Glycated hemoglobin
RCTs: Randomized controlled trials
DCCT: Diabetes Control and Complications Trial
UKPDS: United Kingdom Prospective Diabetes Study
miRs: MicroRNAs
FMD: Flow-mediated dilation
T1DM: Type 1 diabetes mellitus
LEADER: The Liraglutide Effect and Action in Diabetes: Evaluation of Cardiovascular Outcome Results
SUSTAIN-6: Semaglutide in Subjects with Type 2 Diabetes
GLP-1R: Glucagon-like peptide 1 receptor
DPP-4: Dipeptidyl peptidase 4
SGLT2: Sodium-glucose cotransporter 2
SAVOR-TIMI: The Saxagliptin Assessment of Vascular Outcomes Recorded in Patients with Diabetes Mellitus-Thrombolysis in Myocardial Infarction
MACE: Major adverse cardiovascular events
TECOS: Sitagliptin Cardiovascular Outcomes Assessment Trial
CIMT: Carotid intima-media thickness
ACE: Acarbose Cardiovascular Evaluation
TZDs: Thiazolidinediones
PPARs: Peroxisome proliferator-activated receptors
ETB: ET-1 receptor B
PROactive trial: The prospective pioglitazone clinical trial in macrovascular events
AMP: Adenosine 5′-monophosphate
AMPK: Activated protein kinase
TCM: Traditional Chinese medicine
STZ: Streptozotocin
GSA: Glycated serum albumin
NAD: Nicotinamide adenine dinucleotide
Nrf2: Nuclear factor E2-related factor 2
HSP90: Heat shock protein 90
GTPCH1: GTP cyclin-1
DHFR: Dihydrofolate reductase
oxLDL: Oxidized low-density lipoprotein
